# Stratification by Smoking Status Reveals an Association of *CHRNA5-A3-B4* Genotype with Body Mass Index in Never Smokers

**DOI:** 10.1371/journal.pgen.1004799

**Published:** 2014-12-04

**Authors:** Amy E. Taylor, Richard W. Morris, Meg E. Fluharty, Johan H. Bjorngaard, Bjørn Olav Åsvold, Maiken E. Gabrielsen, Archie Campbell, Riccardo Marioni, Meena Kumari, Jenni Hällfors, Satu Männistö, Pedro Marques-Vidal, Marika Kaakinen, Alana Cavadino, Iris Postmus, Lise Lotte N. Husemoen, Tea Skaaby, Tarunveer S. Ahluwalia, Jorien L. Treur, Gonneke Willemsen, Caroline Dale, S. Goya Wannamethee, Jari Lahti, Aarno Palotie, Katri Räikkönen, Aliaksei Kisialiou, Alex McConnachie, Sandosh Padmanabhan, Andrew Wong, Christine Dalgård, Lavinia Paternoster, Yoav Ben-Shlomo, Jessica Tyrrell, John Horwood, David M. Fergusson, Martin A. Kennedy, Tim Frayling, Ellen A. Nohr, Lene Christiansen, Kirsten Ohm Kyvik, Diana Kuh, Graham Watt, Johan Eriksson, Peter H. Whincup, Jacqueline M. Vink, Dorret I. Boomsma, George Davey Smith, Debbie Lawlor, Allan Linneberg, Ian Ford, J. Wouter Jukema, Christine Power, Elina Hyppönen, Marjo-Riitta Jarvelin, Martin Preisig, Katja Borodulin, Jaakko Kaprio, Mika Kivimaki, Blair H. Smith, Caroline Hayward, Pål R. Romundstad, Thorkild I. A. Sørensen, Marcus R. Munafò, Naveed Sattar

**Affiliations:** 1 MRC Integrative Epidemiology Unit (IEU) at the University of Bristol, Bristol, United Kingdom; 2 UK Centre for Tobacco and Alcohol Research Studies, School of Experimental Psychology, University of Bristol, Bristol, United Kingdom; 3 Department of Primary Care and Population Health, UCL, London, United Kingdom; 4 Department of Public Health, Faculty of Medicine, Norwegian University of Science and Technology, Trondheim, Norway; 5 Department of Cancer Research and Molecular Medicine, Norwegian University of Science and Technology, Trondheim, Norway; 6 Medical Genetics Section, Centre for Genomic and Experimental Medicine, Institute of Genetics and Molecular Medicine, University of Edinburgh, Edinburgh, United Kingdom; 7 Centre for Cognitive Ageing and Cognitive Epidemiology, University of Edinburgh, Edinburgh, United Kingdom; 8 Queensland Brain Institute, University of Queensland, Brisbane, Australia; 9 Department of Epidemiology and Public Health, University College London, London, United Kingdom; 10 Institute for Social and Economic Research, University of Essex, Colchester, United Kingdom; 11 Institute for Molecular Medicine Finland FIMM, University of Helsinki, Helsinki, Finland; 12 Department of Public Health, Hjelt Institute, University of Helsinki, Helsinki, Finland; 13 National Institute for Health and Welfare, Helsinki, Finland; 14 Department of Internal Medicine, Internal Medicine, Lausanne University Hospital, Lausanne, Switzerland; 15 Department of Epidemiology and Biostatistics, MRC Health Protection Agency (HPA) Centre for Environment and Health, School of Public Health, Imperial College London, London, United Kingdom; 16 Institute of Health Sciences, University of Oulu, Oulu, Finland; 17 Biocenter Oulu, University of Oulu, Oulu, Finland; 18 Centre for Paediatric Epidemiology and Biostatistics, UCL Institute of Child Health, London, United Kingdom; 19 Department of Gerontology and Geriatrics, Leiden University Medical Center, Leiden, The Netherlands; 20 Netherlands Consortium of Healthy Ageing, Leiden, The Netherlands; 21 Research Centre for Prevention and Health, The Capital Region, Copenhagen, Denmark; 22 Novo Nordisk Foundation Centre for Basic Metabolic Research, Metabolic Genetics Section, Faculty of Health and Medical Sciences, University of Copenhagen, Copenhagen, Denmark; 23 Copenhagen Prospective Studies on Asthma in Childhood, Health Sciences, University of Copenhagen, Copenhagen, Denmark; 24 Danish Pediatric Asthma Center, Copenhagen University Hospital, Gentofte, Denmark; 25 Netherlands Twin Register, Department of Biological Psychology, VU University, Amsterdam, The Netherlands; 26 Faculty of Epidemiology and Population Health, London School of Hygiene and Tropical Medicine, London, United Kingdom; 27 Institute of Behavioural Sciences, University of Helsinki, Helsinki, Finland; 28 Folkhälsan Research Centre, Helsinki, Finland; 29 Wellcome Trust Sanger Institute, Wellcome Trust Genome Campus, Cambridge, United Kingdom; 30 Institute for Molecular Medicine Finland (FIMM), University of Helsinki, Helsinki, Finland; 31 The Medical and Population Genomics Program, The Broad Institute of MIT and Harvard, Cambridge, Massachusetts, United States of America; 32 Robertson Centre for Biostatistics, University of Glasgow, Glasgow, United Kingdom; 33 Institute of Cardiovascular and Medical Sciences, University of Glasgow, Glasgow, United Kingdom; 34 MRC Unit for Lifelong Health and Ageing at UCL, London, United Kingdom; 35 Institute of Public Health, Department of Environmental Medicine, University of Southern Denmark, Odense, Denmark; 36 School of Social and Community Medicine, University of Bristol, Bristol, United Kingdom; 37 European Centre for Environment and Human Health, University of Exeter Medical School, Truro, United Kingdom; 38 Genetics of Complex Traits, University of Exeter Medical School, Exeter, United Kingdom; 39 Department of Psychological Medicine, University of Otago, Christchurch, New Zealand; 40 Department of Pathology, University of Otago, Christchurch, New Zealand; 41 Institute for Clinical Research, University of Southern Denmark, Odense, Denmark; 42 Department of Public Health, Aarhus University, Aarhus, Denmark; 43 Institute of Public Health, Dept. of Epidemiology, Biostatistics and Biodemography, University of Southern Denmark, Denmark; 44 Institute of Regional Health Research, University of Southern Denmark, Odense, Denmark; 45 Odense Patient data Explorative Network (OPEN), Odense University Hospital, Odense, Denmark; 46 Institute of Health and Wellbeing, University of Glasgow, Glasgow, United Kingdom; 47 Department of General Practice and Primary Health Care, University of Helsinki, Helsinki, Finland; 48 Helsinki University Central Hospital, Unit of General Practice, Helsinki, Finland; 49 Vasa Central Hospital, Vasa, Finland; 50 Population Health Research Institute, St George's University of London, London, United Kingdom; 51 Department of Clinical Experimental Research, Glostrup University Hospital, Glostrup, Denmark; 52 Department of Clinical Medicine, Faculty of Health and Medical Sciences, University of Copenhagen, Denmark; 53 Department of Cardiology, Leiden University Medical Center, Leiden, The Netherlands; 54 Durrer Center for Cardiogenetic Research, Amsterdam, The Netherlands; 55 Interuniversity Cardiology Institute of the Netherlands, Utrecht, The Netherlands; 56 School of Population Health and Sansom Institute, University of South Australia, Adelaide, Australia; 57 South Australian Health and Medical Research Institute, Adelaide, Australia; 58 Unit of Primary Care, Oulu University Hospital, Oulu, Finland; 59 Department of Children and Young People and Families, National Institute for Health and Welfare, Oulu, Finland; 60 Department of Psychiatry, Lausanne University Hospital, Lausanne, Switzerland; 61 University of Dundee, Ninewells Hospital and Medical School, Dundee, United Kingdom; 62 Medical Research Council Human Genetics Unit, Institute of Genetics and Molecular Medicine, University of Edinburgh, Edinburgh, United Kingdom; 63 Institute of Preventive Medicine, Bispebrjerg and Frederikberg Hospitals, The Capital Region, Copenhagen, Denmark; 64 BHF Glasgow Cardiovascular Research Centre, Faculty of Medicine, University of Glasgow, Glasgow, United Kingdom; Georgia Institute of Technology, United States of America

## Abstract

We previously used a single nucleotide polymorphism (SNP) in the *CHRNA5-A3-B4* gene cluster associated with heaviness of smoking within smokers to confirm the causal effect of smoking in reducing body mass index (BMI) in a Mendelian randomisation analysis. While seeking to extend these findings in a larger sample we found that this SNP is associated with 0.74% lower body mass index (BMI) per minor allele in current smokers (95% CI -0.97 to -0.51, *P* = 2.00×10^−10^), but also unexpectedly found that it was associated with 0.35% *higher* BMI in never smokers (95% CI +0.18 to +0.52, *P* = 6.38×10^−5^). An interaction test confirmed that these estimates differed from each other (*P* = 4.95×10^−13^). This difference in effects suggests the variant influences BMI both via pathways unrelated to smoking, and via the weight-reducing effects of smoking. It would therefore be essentially undetectable in an unstratified genome-wide association study of BMI, given the opposite association with BMI in never and current smokers. This demonstrates that novel associations may be obscured by hidden population sub-structure. Stratification on well-characterized environmental factors known to impact on health outcomes may therefore reveal novel genetic associations.

## Author Summary

We found that a single nucleotide polymorphism in the *CHRNA5-A3-B4* gene cluster, which is known to influence smoking heaviness, is associated with lower body mass index (BMI) in current smokers, but *higher* BMI in never smokers. This difference in effects suggests that the variant influences BMI both via pathways other than smoking, and via the weight-reducing effects of smoking, in opposite directions. The overall effect on BMI would therefore be undetectable in an unstratified genome-wide association study, indicating that novel associations may be obscured by hidden population sub-structure.

## Introduction

As obesity represents a substantial and growing threat to public health, efforts to identify the determinants of obesity are of considerable scientific and societal importance. Genome-wide association studies (GWAS) have identified numerous variants associated with body mass index (BMI) [1], but a substantial proportion of the estimated heritability remains to be accounted for. At the same time, a number of modifiable environmental factors have been identified that influence BMI, with cigarette smoking a strong lifestyle influence on BMI [2]. In a previous Mendelian randomisation analysis, we used a single nucleotide polymorphism in the *CHRNA5-A3–B4* gene cluster associated with heaviness of smoking within smokers [3] to confirm the causal effect of smoking in reducing BMI [4].

We sought to extend these findings in a larger sample drawn from the Causal Analysis Research in Tobacco and Alcohol (CARTA) consortium (http://www.bris.ac.uk/expsych/research/brain/targ/research/collaborations/carta/). We used the same genetic variant, characterised by two SNPs (rs16969968 and rs1051730) which are in perfect linkage disequilibrium (LD) in samples of European ancestry, and therefore reflect the same genetic signal (hereafter rs16969968-rs1051730). This variant is associated with approximately 1% phenotypic variance in cigarettes per day and approximately 4% variance in cotinine levels (the primary metabolite of nicotine, and a more precise measure of exposure) [5], [6]. Mendelian randomisation analyses of the causal effects of smoking heaviness require stratification according to smoking status – any causal effects of the exposure (i.e., smoking heaviness) should be reflected in an association of the instrument (i.e., genotype) among current smokers only, and not never smokers (former smokers might be expected to be intermediate between current and never smokers) [7]. The never smoking group therefore enables a test of the specificity of the instrument (i.e., that the variant *only* affects the outcome through the exposure of interest) [8]. Critically, the rs16969968-rs1051730 variant has not been shown to be associated with smoking initiation (i.e., it does not influence risk of being an ever versus a never smoker) in previous GWAS of smoking behaviour [9], which reduces the risk of introducing collider bias when stratifying on smoking status.

In the course of these analyses, we observed an unexpected finding, which we report here. Specifically, we observed an association of rs16969968-rs1051730 with *higher* BMI in never smokers. This association has not previously been reported in GWAS of BMI published to date. We therefore focus on the implications of this novel finding, and not the Mendelian randomisation analysis of the causal effects of smoking on BMI.

## Results

Our total sample size comprised 148,730 never smokers, former smokers and current smokers. In the 66,809 never smokers, we observed positive association of rs16969968-rs1051730 with BMI (Table 1), indicating an association operating via pathways other than smoking (percentage change per minor allele +0.35, 95% CI +0.18 to +0.52, P = 6.38×10^−5^). We also confirmed the expected inverse association of rs16969968-rs1051730 with BMI in the 38,913 current smokers (percentage change −0.74, 95% CI −0.97 to −0.51, *P* = 2.00×10^−10^), consistent with a causal, weight-reducing effect of cigarette smoking on BMI. There was no evidence of association in the 43,009 former smokers (percentage change −0.14, 95% CI −0.34 to +0.07, *P* = 0.19). An interaction test indicated that these estimates differed from each other (*P* = 4.95×10^−13^). Similar associations were observed for weight (Table 1) and waist circumference (data available on request), but not height (*P*s ≥0.27 for all smoking categories). Between-study heterogeneity was low (I^2^ values ≤36%), and there was no evidence for effect modification by sex. Critically, when data were examined without stratification by smoking status no clear evidence of association with BMI was observed (*P* = 0.22), indicating that a conventional GWAS would have failed to detect this signal.

**Table 1:** Association of rs16969968-rs1051730 with body mass index, weight and height, stratified by smoking status.

	Sample Size	Effect	95% CI	P-value	P-value (interaction)
**BMI**					
Never Smoker	66,809	0.35	0.18, 0.52	6.38×10^−5^	4.95×10^−13^
Former Smoker	43,009	−0.14	−0.34, 0.07	0.19	
Current Smoker	38,913	−0.74	−0.97, −0.51	2.00×10^−10^	
Total	148,730	−0.07	−0.19, 0.04	0.22	
**Weight**					
Never Smoker	66,809	0.37	0.18, 0.55	9.21×10^−5^	2.76×10^−12^
Former Smoker	43,009	−0.07	−0.30, 0.16	0.55	
Current Smoker	38,913	−0.79	−1.04, −0.54	6.44×10^−10^	
Total	148,730	−0.05	−0.17, 0.08	0.44	
**Height**					
Never Smoker	66,809	0.02	−0.05, 0.09	0.53	0.55
Former Smoker	43,009	0.05	−0.04, 0.14	0.27	
Current Smoker	38,913	−0.02	−0.11, 0.07	0.66	
Total	148,730	0.02	−0.03, 0.07	0.44	

All analyses were adjusted for age. Effect estimate represents per minor allele percentage change for BMI and weight (log transformed for analysis), and per minor allele change in cm for height.

The 0.35% per minor allele BMI increase in never smokers represents a change of approximately 0.09 kg/m^2^. This is smaller than the effect of rs9939609 in *FTO* (∼0.4 kg/m^2^) [10] but is comparable in terms of variance explained to the other variants identified by Speliotes and colleagues [1]. As noted above, the rs16969968-rs1051730 variant has not been shown to be associated with smoking initiation in previous GWAS of smoking behaviour [9]. This is also true in our data (ever smoker versus never smoker: OR per minor allele 1.01, 95% CI 0.99 to 1.03, P = 0.50), although we observed an association with smoking cessation (current smoker versus former smoker: OR per minor allele 1.08, 95% CI 1.06 to 1.10, P = 1.44×10^−12^), consistent with previous studies [11]. Therefore, we do not believe that these findings are due to collider bias, whereby stratifying on the exposure measure can induce associations between instrument and outcome [12].

## Discussion

Our results indicate that rs16969968-rs1051730 may be associated with BMI in never smokers, via pathways other than smoking, as well as with heaviness of smoking among current smokers. At this stage we can only speculate as to the mechanism through which rs16969968-rs1051730 may exert a positive effect on BMI in never smokers. In GWAS, the *CHRNA5-A3-B4* gene cluster was confirmed to be associated with heaviness of smoking, and downstream health outcomes including lung cancer and peripheral arterial disease [9], [13], [14]. It has been shown that the rs16969968 variant is functional and leads to an amino acid change (D398N) in the α5 nicotinic acetylcholine receptor (nAChR) subunit protein [15]. Animal models indicate that this subunit modulates tolerance to high doses of nicotine [16]. Candidate gene studies have suggested an association of rs16969968-rs1051730 with other substance use phenotypes, such as cocaine use [17], while other variants in this region have been reported to be associated with alcohol consumption [18], although the evidence for these associations is currently weak. Therefore, one possibility is that nAChRs play a role in central mechanisms mediating responding to rewarding stimuli in general, which could include natural rewards such as food.

It is also notable that rs3743075, located within the *CHRNA3* gene and correlated with rs16969968-rs1051730 (r^2^ = 0.34, D′ = 1.00), shows association (*N* = 974, *P* = 9.06×10^−5^) with BMI (defined as <30 kg/m^2^ vs ≥30 kg/m^2^) (dbGaP Study Accession: pha003015.1). There is evidence from animal models that activation of hypothalamic α3β4 nAChRs leads to activation of pro-opiomelanocortin neurons, and subsequent activation of melanocortin 4 receptors, which have been shown to be critical for nicotine-induced decreases in food intake [19]. Therefore, another possibility is that nAChR sub-units play a role specifically in mediating food intake, through as yet undescribed mechanisms. In other words, the effects we have observed operate via other nAChRs, and other genes in this region (namely *CHRNA3* and *CHRNB4*) may contribute to our finding. Clearly further work is required to explore this possibility. The use of more detailed body composition measures such as percent body fat and its distribution may also serve to refine the nature of the association.

Our results, if confirmed, have important implications for the design of future GWAS. The association we observed in never smokers would essentially be undetectable in an unstratified sample, since the effect size observed in the combined sample would require approximately 791,000 participants to detect even at an uncorrected *P*-value of 0.05, and even then would indicate an inaccurate effect size. This is essentially because the effect of rs16969968-rs1051730 on BMI that operates via pathways other than smoking is countered by the weight-reducing effect of smoking. Therefore, since there are roughly twice as many never smokers as current smokers on average across our sample, these two effects negate each other. On the other hand, a sample of approximately 160,000 never smokers would be required to detect the effect we observed with genome-wide significance. Assuming the proportions of never, former and current smokers in our sample, this would imply a total sample size of around 350,000. While this is larger than published GWAS of BMI [1], it is achievable. Therefore, although we cannot say how frequent a scenario such as the one we observed here will be, additional variants may be identified in GWAS stratified by environmental exposures known to have pronounced effects on the phenotype of interest, such as cigarette smoking or physical activity on BMI.

The pleiotropic effect of rs16969968-rs1051730 (or LD of this variant with another variant causally influencing BMI), if shown to be robust via replication, has important implications for Mendelian randomisation studies assessing the causal effects of smoking. In this case, we can be reasonably confident that the BMI-reducing effect of the variant operates through smoking because the association with BMI in current smokers is in the opposite direction to the association in never smokers. Furthermore, if the effects on BMI that operate via pathways other than smoking and the effects that operate via the weight-reducing effects of smoking are independent, then the true causal estimate of the magnitude of effect of smoking in reducing BMI is likely to be *larger* than estimated with this variant. However, some caution must be exercised in conducting and interpreting the results of other Mendelian randomisation analyses using this variant because rs16969968-rs1051730 may influence outcomes through its effects on BMI, instead of or in addition to smoking heaviness. One possible solution is to use genetic variants for BMI as a method of reciprocal randomization to determine the direction of causation within inter-correlated networks of mechanistic pathways (i.e., network Mendelian randomisation) [20].

A limitation to our analysis is that we were only able to control for potential population stratification indirectly in most samples, by restricting analyses to participants of self-reported European ancestry. We were not able to use other methods, such as adjustment for principal components, given that not all contributing studies hold the necessary genetic data. However, we note that the minor allele frequency of the rs16969968-rs1051730 differed only slightly across studies (between 0.30 and 0.36).

Testing for gene-environment interaction in GWAS is not novel [21], and examples exist which incorporate smoking status as an environmental factor [22]. However, this remains relatively uncommon, due to methodological challenges (e.g., introducing collider bias) and sample size constraints. A key challenge is the identification of suitable environmental variables on which to stratify GWAS analyses, from the multitude available. We suggest that focusing on environmental factors that are most strongly associated with the phenotype of interest, are likely to have profound biological effects, and which can be characterised in a relatively consistent way across studies, is likely to be the best strategy. Smoking status meets all of these criteria, and the data presented here demonstrate how stratification on well-characterized environmental factors known to impact on health outcomes (such as smoking status) may reveal novel genetic associations with health outcomes. As our data indicate, these associations may operate through genetic influences on the environmental factors themselves, or through new pathways which are masked by the environmental factors.

## Methods

### Study populations

We used data on individuals (≥16 years) of European ancestry (ascertained via self report, or based on the genome-wide genotype data where available) from 29 studies in the Causal Analysis Research in Tobacco and Alcohol (CARTA) consortium (http://www.bris.ac.uk/expsych/research/brain/targ/research/collaborations/carta/): the 1958 Birth Cohort (1958 BC), the Avon Longitudinal Study of Parents and Children (ALSPAC, including both mothers and children), the British Regional Heart Study (BRHS), the British Women's Heart and Health Study (BWHHS), the Caerphilly Prospective Study (CaPS), the Christchurch Health and Development Study (CHDS), the Cohorte Lausannoise (CoLaus) study, the Exeter Family Study of Child Health (EFSOCH), the English Longitudinal Study of Ageing (ELSA), FINRISK, the Danish GEMINAKAR twin study, Generation Scotland, the Genomics of Overweight Young Adults (GOYA) females, GOYA males, the Helsinki Birth Cohort Study (HBCS), Health2006, Health2008, the Nord-Trøndelag health study (HUNT), Inter99, the Northern Finland Birth Cohorts (NFBC 1966 and NFBC 1986), MIDSPAN, the Danish MONICA study, the National Health and Nutrition Examination Survey (NHANES), the MRC National Survey of Health & Development (NSHD), the Netherlands Twin Registry (NTR), the Prospective Study of Pravastatin in the Elderly at Risk (PROSPER) and Whitehall II. References to these individual studies are available on request. All studies received ethics approval from local research ethics committees (see Text S1 for full details).

### Genotyping

Within each study, individuals were genotyped for one of two single nucleotide polymorphisms (SNPs) in the *CHRNA5-A3-B4* nicotinic receptor subunit gene cluster, rs16969968 or rs1051730. These single nucleotide polymorphisms are in perfect linkage disequilibrium with each other in Europeans (R^2^ = 1.00 in HapMap 3, http://hapmap.ncbi.nlm.nih.gov/) and therefore represent the same genetic signal. Where studies had data available for both SNPs, we used the SNP that was genotyped in the largest number of individuals.

### Body mass index

Height (m), weight (kg) and waist circumference (cm) were assessed within each study, directly measured for 99% of participants, and self-reported for GOYA females (N = 1,015) and a sub-set of NTR (N = 602). Body mass index (BMI) was calculated as weight/height^2^.

### Smoking status

Smoking status was self-reported (either by questionnaire or interview). Individuals were classified as current, former, or never cigarette smokers. Where information on smoking frequency was available, current smokers were restricted to individuals who smoked regularly (typically at least one cigarette per day). Where information on pipe and cigar smoking was available, individuals reporting being current or former smokers of pipes or cigars but not cigarettes were excluded from all analyses. For studies with adolescent populations (ALSPAC children and NFBC 1986), analyses were restricted to current daily smokers who reported smoking at least one cigarette per day (current smokers) and individuals who had never tried smoking (never smokers). Descriptive characteristics of smoking frequency data are provided in Text S2.

### Statistical analysis

Analyses were conducted within each contributing study using Stata and R software, following the same analysis plan. Analyses were restricted to individuals with full data on smoking status and rs16969968-rs1051730 genotype. Within each study, genotype frequencies were tested for deviation from Hardy Weinberg Equilibrium (HWE) using a chi-squared test. Mendelian randomisation analyses of the association between rs16969968-rs1051730 and BMI were performed using linear regression, stratified by smoking status (never, former and current) and sex, and adjusted for age. BMI was log transformed prior to analysis. An additive genetic model was assumed on log values, so that each effect size could be exponentiated to represent the percentage increase in BMI per minor (risk) allele.

For NHANES, which has a survey design, Taylor series linearization was implemented to estimate variances. For studies including related family members appropriate methods were used to adjust standard errors: in GEMINAKAR, twin pair identity was included as a cluster variable in the model, in MIDSPAN linear mixed effects regression models fitted using restricted maximum likelihood were used to account for related individuals. ALSPAC mothers and children were analysed as separate samples; as there are related individuals across these samples, sensitivity analyses were performed excluding each of these studies in turn.

Results from individual studies were meta-analysed in Stata (version 13) using the “metan” command. As I^2^ values were all equal to or below 36% (indicating low to moderate heterogeneity), fixed effects meta-analyses were performed. The “metareg” command was used to examine whether SNP effects varied by sex and estimates were combined as there was no evidence for effect modification by sex. Evidence for interaction between genotype and smoking status was assessed using the Cochran Q statistic. Data are available from the Institutional Data Access/Ethics Committees of the individual studies that contributed to this analysis, for researchers who meet the criteria for access to confidential data. Full details are provided in Text S3.

### Sample size calculations

Sample size calculations were performed using Quanto software (http://biostats.usc.edu/Quanto.html). The following parameters were used: 80% power to detect associations, minor allele frequency of 0.33, mean and standard deviation for BMI of 25 kg/m^2^ and 3.8 kg/m^2^ respectively, alpha values of 0.05 and 5×10^−8^.

## Supporting Information

Text S1 Ethics approvals for individual contributing studies.
(DOCX)

Text S2 Smoking heaviness in the CARTA studies.
(DOCX)

Text S3 Data access arrangements for individual contributing studies.
(DOCX)
