# Supplementary material for: Stratification by Smoking Status Reveals an Association of CHRNA5-A3-B4 Genotype with Body Mass Index in Never Smokers
Source: PLoS Genet. 2014 Dec 4;10(12):e1004799. doi: 10.1371/journal.pgen.1004799 (PMC4256159; doi:10.1371/journal.pgen.1004799)
Supplement: Text S1 — Ethics approvals for individual contributing studies. (DOCX) [file pgen.1004799.s001.docx]

**Text S1. Ethics approvals for individual contributing studies.**

**1958BC**

The 45-year biomedical survey and genetic studies were approved by the South-East Multi- Centre Research Ethics Committee (01/1/44) and the joint UCL/UCLH Committees on the Ethics of Human Research (08/H0714/40).

**ALSPAC**

Ethics approval for the study was obtained from the ALSPAC Ethics and Law Committee and the Local Research Ethics Committee. The study website contains details of all the data that is available through a fully searchable data dictionary (<http://www.bristol.ac.uk/alspac/researchers/data-access/data-dictionary/>).

**BRHS**

The BRHS has local (from each of the districts in which the study was based) and multi-centre ethical committee approvals.

**BWHHS**

Ethics approval was granted for the BWHHS from the London Multi-Centre Research Ethics Committee and 23 Local Research Ethics Committees.

**CaPS**

Ethics approval was obtained from the MRC Epidemiology Unit (Cardiff), the South Glamorgan Area Health Authority, the Gwent REC, and the South Wales Research Ethics Committee D, and each subject signed their agreement to be involved.

**CHDS**

All phases of the study have received ethics approval from the regional Health and Disability Ethics Committee and all forms of data collection have been subject to the signed consent of research participants.

**Colaus/ PsyCoLaus**

Colaus and PsyCoLaus were approved by the Institutional Ethics Committee of the University of Lausanne.

**Dan-MONICA10**

The Dan-MONICA10 was approved by the local ethics committee of Copenhagen County and all participants gave their signed informed consent.

**EFSOCH**

Ethics approval was given by the North and East Devon Local Research Ethics Committee.

**ELSA**

ELSA has been approved by the National Research Ethics Service and all participants have given informed consent.

**FINRISK**

The 2002 and 2007 FINRISK surveys have been approved by the Coordinating Ethics Committee of the Helsinki University Hospital District. Each participant has given a written informed consent.

**GEMINAKAR**

The study was approved by the relevant Danish Ethics Committee (baseline, S-VF-19970271) and Danish Data Protection Board (baseline, 1999-1200-441). All participants provided written informed consent.

**Generation Scotland**

Ethics approval for the study was given by the NHS Tayside committee on research ethics (05/s1401/89).

**GOYA**

The study was approved by the regional scientific ethics committee and by the Danish Data Protection Board.

**HBCS**

The research plan of the HBCS was approved by the Institutional Review Board of the National Public Health Institute and all participants have signed an informed consent.

**Health2006/Health2008/Inter99**

The studies have been approved by the Ethical Committee of Copenhagen.

**HUNT**

Use of data in the present study was approved by the Regional Committee for Medical Research Ethics (2013/1127/REK midt).

**Midspan**

Ethics approval was obtained from the Argyll and Clyde Health Board Local Research Ethics

**NFBC**

The University of Oulu Ethics Committee and the Ethical Committee of Northern Ostrobothnia Hospital District have approved the study. Participants provided written informed consent.

**NHANES**

Data collection for NHANES was approved by the NCHS Research Ethics Review Board. Analysis of de-identified data from the survey is exempt from the federal regulations for the protection of human research participants. Analysis of restricted data through the NCHS Research Data Center is also approved by the NCHS ERB. The findings and conclusions in this paper are those of the author(s) and do not necessarily represent the views of the Research Data Center, the National Center for Health Statistics, or the Centers for Disease Control and Prevention.

**NSHD**

Ethics approval was given by the Central Manchester Research Ethics Committee.

**NTR**

The NTR study was approved by the Central Ethics Committee on Research Involving Human Subjects of the VU University Medical Center, Amsterdam (IRB number IRB-2991 under Federalwide Assurance 3703; IRB/institute code 03-180), and all subjects provided written informed consent.

**PROSPER**

PROSPER was approved by the Argyll and Clyde Local Research Ethics Committee, the Glasgow Royal Infirmary Local Research Ethics Committee, Greater Glasgow Primary Care and Mental Health Research Ethics Committee, Lanarkshire Health Board Local Research Ethics Committee, Dumfries and Galloway Health Board Local Research Ethics Committee, Forth Valley Health Board Local Research Ethics Committee, METC board of Leiden University Medical Center and the Clinical Research Ethics Committee of The Cork Teaching Hospitals, and all participants gave written informed consent.

**Whitehall**

Ethics approval for the Whitehall II study was obtained from the University College London Medical School committee on the ethics of human research. Informed consent was gained from every participant.
