# Supplementary material for: Stratification by Smoking Status Reveals an Association of CHRNA5-A3-B4 Genotype with Body Mass Index in Never Smokers
Source: PLoS Genet. 2014 Dec 4;10(12):e1004799. doi: 10.1371/journal.pgen.1004799 (PMC4256159; doi:10.1371/journal.pgen.1004799)
Supplement: Text S2 — Smoking heaviness in the CARTA studies. (DOCX) [file pgen.1004799.s002.docx]

**Text S2. Smoking heaviness in the CARTA studies.**

| **Study** | **N**  **(current smokers)** | **N**  **(with cig/day data)** | **Cigarettes per day (continuous)** | | | | **Cigarettes per day (categorical)** |
| --- | --- | --- | --- | --- | --- | --- | --- |
|  |  |  | **Mean** | **SD** | **Minimum** | **Maximum** | **N per category** |
| 1958 BC | 1,220 | 1,220 | 17.2 | 8.3 | 1 | 70 |  |
| ALSPAC Children | 214 | 214 | 8.8 | 5.8 | 1 | 30 |  |
| ALSPAC Mothers | 131 | 123 | 11.3 | 6.6 | 1 | 40 |  |
| BRHS | 464 | 405 | 13.8 | 9.1 | 0 | 60 |  |
| BWHHS | 392 | 392 |  |  |  |  | **1-9**: 155, **10-19**: 165, **20-29**: 62, **30+:** 10 |
| CaPS | 337 | 337 |  |  |  |  | **1-14:** 164, **15-24:** 117, **25+:** 56 |
| CHDS | 165 | 165 |  |  |  |  | **1-9:** 66, **10-19:** 80, **20+:** 19 |
| CoLaus | 1,095 | 1,062 | 16.9 | 11.1 | 1 | 60 |  |
| Dan-MONICA | 1,028 | 909 | 16.2 | 7.4 | 1.0 | 60 |  |
| EFSOCH | 238 | 238 |  |  |  |  | **1-4:** 60, **5-9:**47, **10-14:** 49, **15-19:** 40, **20-24:** 35, **25-29:** 5, **30+:** 2 |
| ELSA | 701 | 522 | 13.9 | 8.2 | 1 | 50 |  |
| FINRISK | 5,120 | 4,920 | 16.5 | 8.2 | 1 | 100 |  |
| GEMINAKAR | 379 | 379 | 12.6 | 6.9 | 0 | 40 |  |
| Generation Scotland | 980 | 980 |  |  |  |  | **0-4:** 162, **5-9:** 117, **10-14:** 196, **15-19:** 149, **20-24:** 248, **25-29:** 46, **30-34:** 32, **35-39:** 10, **40-44:** 13, **45-49**: 1, **50+:** 6 |
| GOYA females | 148 | 148 |  |  |  |  | **1-9**: 76, **10-19:** 66, **20+:** 6 |
| GOYA males | 380 | 308 | 17.5 | 10.6 | 1 | 60 |  |
| HBCS | 372 | 372 |  |  |  |  | **1-5:** 75, **6-20:** 221, **21+:** 76 |
| Health2006 | 751 | 713 | 15.7 | 7.8 | 1 | 50 |  |
| Health2008 | 123 | 119 | 14.6 | 7.4 | 2 | 40 |  |
| HUNT | 17,030 | 16,322 | 11.2 | 5.7 | 1 | 70 |  |
| Inter99 | 1,986 | 1,875 | 17.5 | 8.2 | 1 | 80 |  |
| NFBC1966 | 1,389 | 1,389 | 14.7 | 7.0 | 1 | 50 |  |
| NFBC1986 | 419 | 373 | 9.2 | 5.9 | 1 | 50 |  |
| MIDSPAN | 531 | 531 | 16.6 | 8.9 | 1 | 50 |  |
| NHANES | 442 | Information not available | | | | | |
| NSHD | 336 | 332 | 15.7 | 9.8 | 1 | 60 |  |
| NTR | 815 | 597 | 11.9 | 7.5 | 0.05 | 50 |  |
| PROSPER | 1,362 | Data not collected | | | | | |
| Whitehall II | 365 | 329 | 16.2 | 10.1 | 1 | 40 |  |
