# Supplementary material for: Stratification by Smoking Status Reveals an Association of CHRNA5-A3-B4 Genotype with Body Mass Index in Never Smokers
Source: PLoS Genet. 2014 Dec 4;10(12):e1004799. doi: 10.1371/journal.pgen.1004799 (PMC4256159; doi:10.1371/journal.pgen.1004799)
Supplement: Text S3 — Data access arrangements for individual contributing studies. (DOCX) [file pgen.1004799.s003.docx]

**Text S3. Data access arrangements for individual contributing studies.**

**1958BC**

The 1958 birth cohort data can be accessed via the UK Data Service (<http://ukdataservice.ac.uk/>).

**ALSPAC**

Data used for this submission will be made available on request to the ALSPAC executive committee (alspac-exec@bristol.ac.uk). The ALSPAC data management plan (available here: http://www.bristol.ac.uk/alspac/researchers/data-access/) describes in detail the policy regarding data sharing, which is through a system of managed open access.

**BRHS**

The collection and management of data over the last 34 years of the BRHS has been made possible through grant funding from UK government agencies and charities. We welcome proposals for collaborative projects and data sharing (http://www.ucl.ac.uk/pcph/research-groups-themes/brhs-pub). For general data sharing enquiries, please contact Lucy Lennon ([l.lennon@ucl.ac.uk](mailto:l.lennon@ucl.ac.uk)).

**BWHHS**

All BWHHS data collected is held by the research team based at London School of Hygiene and Tropical Medicine, for ongoing analysis. If you would like to collaborate with the BWHHS team, contact the study coordinator, Antoinette Amuzu (antoinette.amuzu@lshtm.ac.uk) Data and biological samples provided to the collaborators can only be used for the purposes originally stated and must not be used in any other way without re-application to the BWHHS team. No data should be passed on to any third party unless they were specified in the original application.

**CaPS**

Data used for the Caerphilly Prospective study (CaPS) was made available by the CaPS access committee (Chair: Professor Kay Tee Khaw). More infomation about its managed access procedure is available on the study website (http://www.bris.ac.uk/social-community-medicine/people/project/1392).

**CHDS**

Data contributed for this submission are available on request from the CHDS (john.horwood@otago.ac.nz)

**CoLaus**

Data from the CoLaus/PsyCoLaus study can be requested according to the procedure described on the CoLaus website (http://www.colaus.ch/en/cls_home/cls_pro_home/cls-research-3.htm).

**EFSOCH**

Summary statistics from the EFSOCH study that contributed to this meta-analysis are available upon request from Jessica Tyrrell (j.tyrrell@exeter.ac.uk)

**ELSA**

ELSA data are made available through the ESDS website (http://www.elsa-project.ac.uk/availableData).

**FINRISK**

Data used for this submission will be made available on request to the FINRISK Management Group, according to the given ethical guidelines and Finnish legislation

**GEMINAKAR**

Data used for this submission will be made available on request to the GEMINAKAR/DTR executive committee (tvilling@health.sdu.dk). The GEMINAKAR/DTR data management plan (http://www.sdu.dk/en/Om_SDU/Institutter_centre/Ist_sundhedstjenesteforsk/Centre/DTR/Researcher) describes in detail the policy regarding data sharing.

Generation Scotland Generation Scotland (GS) data is available on request (access@generationscotland.org). GS has Research Tissue Bank status, and the GS Access Committee reviews applications to ensure that they comply with legal requirements, ethics and patient consent.

**GOYA males**

Access to the original data used in the present study in anonymized form is granted by an application to the study PI, Professor Thorkild IA Sørensen (TSOE0005@regionh.dk).

**GOYA females**

An anonymized copy of the data used for this submission will be made available on request to the GOYA analysts after permission have been given by the DNBC executive committee (www.dnbc.dk).

**HBCS**

Data used for this submission will be made available on request to the HBCS executive committee ([johan.eriksson@helsinki.fi](mailto:johan.eriksson@helsinki.fi)).

**Health2006**

Data used for this submission can be made available on request to the Research Centre for Prevention and Health (http://www.regionh.dk/fcfs/Menu/). Please contact Lise Lotte Husemoen (lise.lotte.nystrup.husemoen@regionh.dk) or Allan Linneberg (allan.linneberg@regionh.dk).

**Health2008**

Data used for this submission can be made available on request to the Research Centre for Prevention and Health (http://www.regionh.dk/fcfs/Menu/). Please contact Lise Lotte Husemoen (lise.lotte.nystrup.husemoen@regionh.dk) or Allan Linneberg (allan.linneberg@regionh.dk).

**HUNT**

Data used from the HUNT Study for this submission will be made available on request to the HUNT Data Access Committee (hunt@medisin.ntnu.no). The HUNT data access information (http://www.ntnu.edu/hunt/data) describes in detail the policy regarding data availability.

**Inter99**

Data used for this submission can be made available on request to the Research Centre for Prevention and Health (http://www.regionh.dk/fcfs/Menu/). Please contact Lise Lotte Husemoen (lise.lotte.nystrup.husemoen@regionh.dk) or Allan Linneberg (allan.linneberg@regionh.dk).

**Midspan**

The Midspan data are available to experienced teams of researchers who have applied and been granted permission by the Midspan Steering Committee to use the data for a specific research proposal. Work will be conducted in collaboration with the Midspan team. Further details on how to apply to use data can be found here: (http://www.gla.ac.uk/researchinstitutes/healthwellbeing/research/publichealth/midspan/research/procedure/)

**Monica**

Data used for this submission can be made available on request to the Research Centre for Prevention and Health (http://www.regionh.dk/fcfs/Menu/). Please contact Lise Lotte Husemoen (lise.lotte.nystrup.husemoen@regionh.dk) or Allan Linneberg (allan.linneberg@regionh.dk).

**NFBC**

Data used for this submission can be made available on request to Tuula Ylitalo (tuula.ylitalo@oulu.fi), Minna Mannikko (minna.annikko@oulu.fi) or Marjo-Riitta Jarvelin (m.jarvelin@imperial.ac.uk).

**NHANES**

NHANES data can be accessed here: (http://www.cdc.gov/nchs/nhanes.htm). The genotype used in this analysis is a restricted variable. Applications for access to these data must be made through the Research Data Center: (http://www.cdc.gov/rdc/).

**NSHD**

The NSHD data are made available to researchers who submit data requests (tomrclha.swiftinfo@ucl.ac.uk). More information is available in the full policy documents (http://www.nshd.mrc.ac.uk/data.aspx). Managed access is in place for this study to ensure that use of the data are within the bounds of consent given previously by participants, and to safeguard any potential threat to anonymity since the participants are all born in the same week.

**NTR**

Data used for this submission will be made available on request to the NTR committee (ntr@psy.vu.nl).

**PROSPER**

The PROSPER study participates in a number of data sharing collaborations and welcomes new well founded proposals. Any proposals would be reviewed by the Study Steering Committee, currently chaired by Wouter Jukema (j.w.jukema@lumc.nl), with respect to scientific validity, feasibility and resource requirements. Any sharing of individual participant data would be dependent on ethical and data privacy constraints and would require a legal data transfer agreement.

**SYS**

Data used for this submission will be made available on request to the SYS principal investigators (http://www.saguenay-youth-study.org) (scientific community/collaborative interests).

**Whitehall II**

Data from the Whitehall II study are made publicly available as described in the Whitehall II data sharing policy (<http://www.ucl.ac.uk/whitehallII/datasharing>).
